# Broken Wires, Dropped Devices, Stuck Stents, Lost Coils and rings! Surgery for device related complications of PCI

**DOI:** 10.1186/1749-8090-10-S1-A94

**Published:** 2015-12-16

**Authors:** Jayakrishnan A Gosalakkal, Suraj Chavan

**Affiliations:** 1 Dept. of Cardiothoracic Surgery, Omega Hospital, Mangalore, 575002, India

## Background/Introduction

Percutaneous interventions for cardiac disease are on the increase worldwide. Acute complications of interventions include vessel dissection, total occlusion, thrombosis etc. Other than the complications associated with the disease process and the disturbances to the vascular endothelium mechanical complications due to mishandling of or mishaps with the device itself can occur. These complications, though rare do occur and the cardiac surgeon has to deal with them often on an emergency basis.

## Aims/Objectives

To assess the safety and efficacy of emergent surgery in patients who develop device related complications of PCI.

## Method

We present 4 patients with coronary artery disease who had complications during angiogram and attempted angioplasty/stenting. In 1 patients the stent was inappropriately deployed and the patient required urgent surgery for stent retrieval. In one the ring tip of the angiography catheter dislodged in the coronary artery requiring emergency surgery and in a further two patients the guide wire broke in the circulation and had to be retrieved along with coronary artery bypass grafting

In another group of 3 patients there was mishandling of the ASD device requiring urgent retrieval and ASD closure. Devices were" lost" in the LA, in the RA and the other in the Pulmonary artery

In 1 patient undergoing device closure of PDA the device was deployed before proper positioning and was brought down to the femoral artery using a bronchoscopic biopsy forceps and then retrieved surgically. In a further patient a PDA coil was "lost' in pulmonary artery, lodging in a small branch. The child underwent surgical closure of PDA. The coil could not be retrieved.

## Results

In conclusion, urgent surgery is often required for device related complications of percutaneous devices. Surgery can be safely undertaken in these patients with gratifying results.

## Discussion/Conclusion

Emergent surgery is lifesaving in patients with device related complications of PCI and can be safely carried out

